# Success in Gingival Recession Coverage: Prognostic Indicators From Private Practice

**DOI:** 10.1155/ijod/7503860

**Published:** 2026-02-18

**Authors:** Michael Saminsky, Liat Chaushu, Benjamin R. Coyac, Alon Sebaoun

**Affiliations:** ^1^ Department of Periodontology and Oral Implantology, Goldschleger School of Dental Medicine, Gray Faculty of Medical and Health Sciences, Tel Aviv University, Tel Aviv, Israel, tau.ac.il; ^2^ Department of Oral Biology, Goldschleger School of Dental Medicine, Gray Faculty of Medical and Health Sciences, Tel Aviv University, Tel Aviv, Israel, tau.ac.il

**Keywords:** gingival recession defects, private practice, risk indicators, root coverage

## Abstract

**Purpose:**

To identify parameters associated with successful root coverage, defined as 70% and 50% root coverage in Miller Class I–II/RT1 and III/RT2 defects, respectively, within periodontal office settings.

**Methods:**

Dental records of patients treated for gingival recessions were screened retrospectively. General health parameters, smoking status, pre and posttreatment recession depth (RD), that is, RD and residual RD (RRD), respectively, recession width (RW), keratinized tissue (KT) width, and clinical attachment loss (CAL) were extracted. Logistic regression linear mixed‐effects models were used to identify correlations between patient‐ and treatment‐specific variables and the success of gingival recession defect coverage up to 180 months.

**Results:**

Records from 105 surgically treated recessions (82 Miller Class‐I–II/RT1, 23 Miller Class‐III/RT2) in 56 patients (46 females, 10 males) were retrieved. Mean follow‐up was 3.52 (1–15) years post‐surgery. Smoking, Miller Class II/RT1 and III/RT2, and lateral teeth were negatively associated with treatment success (effect sizes: smoking −21.0, Miller II/RT1 −21.5, Miller III/RT2 −21.4, lateral −21.1; all *p*  < 0.001), whereas canines and first premolars were positively associated with successful coverage (effect sizes: canine 1.71, first premolar 10.31; all *p*  < 0.001). Univariate linear regression revealed that initial RD, RW, and Miller Class III/RT2 were significantly associated with higher CAL gain (estimates: RD 0.71, RW 0.65, Miller III/RT2 1.14), higher KT gain (estimates: RD 0.34, RW 0.33, Miller III/RT2 0.99), and lower RRD (estimates: Miller II/RT1 0.72, Miller III/RT2 0.92, RD 0.28, RW 0.30) (all *p*  < 0.05).

**Conclusions:**

Long‐term root coverage is negatively correlated with smoking, Miller Class II–III/RT1–2 recessions, and lateral teeth but positively related to canines and first premolars. CAL/KT gain and RRD are positively influenced by initial RD, RW, and Miller Class‐III/RT2.

## 1. Introduction

### 1.1. Etiology of Recession Defects and Treatment Goals

Gingival recession is defined as the apical displacement of the gingival margin from the cementoenamel junction (CEJ), resulting in exposure of the root surface to the oral environment [1]. Its etiology is multifactorial and includes mechanical trauma from improper toothbrushing techniques [2], unfavorable orthodontic tooth movement [3], thin periodontal phenotype, and chronic gingival inflammation [4]. When left untreated, recession defects tend to progress over time, potentially leading to dentin hypersensitivity, root caries, non‐carious cervical lesions, and compromised esthetics [5].

To address these functional and esthetic concerns, a wide range of periodontal plastic surgical techniques have been introduced, aiming to achieve predictable root coverage while restoring a natural and harmonious soft tissue architecture [6]. Contemporary treatment goals extend beyond mere coronal advancement of the gingival margin and include long‐term stability of the results, keratinized tissue gain (KTG), and patient satisfaction with esthetic outcomes [7]. In this context, restorative approaches have also been explored; notably, Class V restorations that increase cervical thickness have been shown to promote soft tissue volume gain, suggesting a potential adjunctive role in achieving optimal soft tissue outcomes [8].

### 1.2. What Constitutes Successful Root Coverage?

Traditionally, the success of root coverage procedures has been assessed using clinician‐centered outcomes such as mean root coverage (MRC) and complete root coverage (CRC). A systematic review by Cairo et al. [9] reported a MRC of approximately 70% and CRC rates of 35% following treatment of Miller Class I and II recession defects. These findings support the general expectation that CRC can be predictably achieved in Class I and II recessions.

However, outcomes for more advanced defects remain less predictable. Chambrone and Tatakis [10] reported MRC ranging from 55% to 85% for Miller Class III or Cairo recession Type 2 defects [10, 11], with CRC achieved in approximately 38% of cases [12, 13]. Consequently, partial root coverage is often considered a successful outcome for these defects. Importantly, such literature‐based thresholds may not fully reflect individual clinical success, particularly when long‐term stability and patient‐centered outcomes are considered.

### 1.3. Variables Influencing Root Coverage Outcomes and Limitations of Current Evidence

Several patient‐related, anatomical, and technique‐related factors have been proposed to influence the success of root coverage procedures, including smoking status, recession depth (RD) and width (RW), periodontal phenotype, keratinized tissue (KT) width, tooth position, and surgical approach [14]. While randomized controlled trials are essential for establishing efficacy, their restrictive inclusion criteria may limit generalizability to daily clinical practice.

Retrospective studies conducted in real‐world clinical settings can provide complementary evidence by capturing long‐term outcomes across a broader spectrum of patients, defect morphologies, and treatment modalities [15–17]. Nevertheless, existing retrospective data remain limited, particularly with respect to long‐term follow‐up, the simultaneous evaluation of multiple clinical and anatomical variables, and the use of advanced statistical models that account for clustering of multiple recession defects within the same patient.

Furthermore, although flap‐based techniques, with or without the addition of a connective tissue graft, are widely used in clinical practice and supported by substantial evidence [18–23], their long‐term performance and prognostic indicators in private practice settings have not been sufficiently explored.

### 1.4. Purpose of the Study

This retrospective study aimed to identify the key clinical, anatomical, and patient‐related (e.g., demographic and lifestyle habits) factors that influence the long‐term success of root coverage procedures in a private practice setting, using logistic regression and linear mixed‐effects models.

## 2. Materials and Methods

The Population, Intervention (Exposure), Comparison, and Outcomes (PI(E)CO) framework was used to define the research question and to investigate the factors influencing the success of root coverage procedures.

### 2.1. Population (P)

The dental records of patients attending a private periodontal practice were retrospectively screened. Patients presenting between 2005 and 2015 with gingival recession defects were examined and treated by the same periodontist (A.S.) and followed up until 2021. Patients seeking root coverage therapy were clinically and radiographically examined, diagnosed, and received a treatment plan that included initial periodontal therapy consisting of oral hygiene instructions, motivation, and scaling. Patients presenting with concomitant caries, defective restorations, or endodontic pathology completed the required dental treatment prior to periodontal surgery.

### 2.2. Inclusion and Exclusion Criteria

Patient files were included only after confirming that all the following data were available:

• General data: sex, age, and smoking status (yes/no) both preoperatively and at follow‐up visits.
• Dental data: tooth number and jaw location (maxilla/mandible).
• Gingival recession data:
  – Recession severity: classified according to Miller (Class I, II, III) and Cairo (RT1, RT2, RT3).
  – Midfacial probing depth (PD).
  – RD: distance from the midfacial CEJ to the most apical point of the free gingival margin.
  – Recession width (RW): tangent drawn at the most apical point of the CEJ between the mesial and distal free gingival margins. When the CEJ was undetectable, the contralateral homologous tooth or adjacent teeth were used to estimate the CEJ level [17].
  – KT width: vertical distance between the midfacial free gingival margin and the alveolar mucosa.
  – Clinical attachment loss (CAL): calculated as the sum of PD and RD.

Patients with systemic conditions, active periodontal disease, or incomplete medical or dental records were excluded from the analysis.

### 2.3. Intervention/Exposure (I/E)

Patients were treated using well‐documented periodontal plastic surgical techniques, specifically flap‐based procedures with or without free connective tissue grafts [18–23]. Postoperative care included suture removal at 14 days, followed by weekly supragingival plaque removal for 8 weeks. Bimonthly follow‐up visits were conducted up to 6 months postsurgery. Each visit included plaque control, bleeding on probing assessment, and reinforcement of oral hygiene motivation. All patients were followed for a minimum of 1 year postoperatively.

### 2.4. Comparison (C)

Given the retrospective observational design, no untreated or alternative‐treatment control group was included. Comparisons were made between baseline (preoperative) and final follow‐up clinical measurements. Based on previously reported outcomes, treatment was considered unsuccessful if root coverage was <70% of the initial RD for Miller Class I/II defects or <50% for Miller Class III defects [9, 10].

### 2.5. Outcomes (O)

Postsurgical final measurements were performed by the same operator (AS) using a UNC‐15 periodontal probe (Hu‐Friedy) and recorded to the nearest millimeter. The following outcome measures were assessed:

• Residual RD (RRD)
• KTG
• Clinical attachment level gain (CAL gain)

Outcome values were calculated following completion of the follow‐up period based on direct comparisons between preoperative and final visit measurements. As this was a retrospective study, formal intra‐examiner calibration was not performed; however, all measurements were obtained by a single experienced clinician, thereby minimizing variability and potential inconsistencies associated with multiple examiners.

### 2.6. Ethics Committee Approval and Patients’ Consent

Patients signed an informed consent, which included the information that the expected results for root coverage were between 70% and 100% of RD when the treated recession was graded Miller I/II, and 50% for a Cl III defect. The signature also signified acceptance that the data could be used for future research. Data were anonymized, and the study protocol was reviewed and approved by the Tel‐Aviv University Ethics Committee (Ref. 008620‐4). The methodology of this study complies with the STROBE (Strengthening the Reporting of Observational Studies in Epidemiology) guidelines/checklist.

### 2.7. Statistical Analysis

Data were tabulated in a computerized database (Microsoft Office Excel Version 14.0, Microsoft Corporation, Redmond, WA, USA). Statistical analysis was performed using Statistics Products Solutions Services (SPSS) 28.0 software for Windows (IBM North America, New York, NY, USA). For minimal sample size calculation, we assumed that the ratio between the number of patients with RT2 to patients with RT1 was 0.45, and that the proportion of successes in the RT1 group was 0.9, and that the proportion of success in the RT2 group was 0.6. Then, for a one‐sided Fisher’s exact test, test significance 0.05, and power 0.8, a sample of 59 independent teeth was found to be required.

Because 21 patients contributed more than one recession site, we fit a logistic regression mixed‐effects model with a random intercept for patient to account for the within‐patient correlation among sites. All mixed‐effects modeling was conducted in R Statistical Software (v4.2.2; R Core Team 2021). Two‐tailed *p*‐values of 0.05 or less were considered statistically significant. *p*‐values were corrected for multiple tests using the Benjamini–Hochberg (BH) method.

## 3. Results

### 3.1. Demographic Analysis

In this retrospective cohort study, a total of 105 recessions were treated in 56 patients comprising 10 males and 46 females with mean ages of 30.7 (18–43) and 31.7 (17–54), respectively. Patients who reported smoking at initial examination did not change their smoking habits throughout the follow‐up period. Oral hygiene was maintained in all patients at levels lower than 20% full mouth plaque and bleeding indices. Thirty‐three patients had single treated recessions, while 21 patients had multiple treated recessions (20 and 1 patients with two and three adjacent recessions, respectively), and 2 patients were treated for both multiple and single recessions. RD ranged between 2 and 10 mm, with 82 Miller Class I–II/RT1 and 23 Miller Class III/RT2 defects (Table 1). The distribution of treated recession defects according to Miller Class/recession type and surgical technique is presented in Table 2. The mean follow‐up was 42 months (range 1–15 years). The most frequently treated teeth were mandibular first incisors, which represent 39% (*n* = 41) of all treated teeth.

**Table 1:** Patient‐related data.

Category	*n*
Patient related‐	
Sex–female; male	46; 10
Age–female; male (range)	31.7 years (17–54); 30.7 years (18–43)
Smoking status (yes; no)	4; 52

Teeth related‐	
Baseline RD range	2–10 mm
Miller Class/RT (*n* teeth)	Miller Cl I–II/RT1–82 Miller Cl III/RT2–23
Follow‐up period–*n* patients (teeth)	1–4 years–42 (79) 5–9 years–11 (19) ≥10 years–3 (7)

**Table 2:** Distribution of treated gingival recession defects according to Miller Class/recession type (RT) and surgical technique.

Total (*n* teeth)	Miller I–II/RT1 (*n* teeth)	Miller III/RT2 (*n* teeth)	Surgical technique
43	32	11	CTG + partial thickness pedicle graft
29	23	6	MTDP
9	7	2	Tunnel technique with simultaneous root coverage and papilla reconstruction
16	16	–	CTG
4	4	–	CAF + CTG
4	–	4	Bilateral pedicle flap‐tunnel technique
105	82	23	–

Abbreviations: CAF, coronally advanced flap; CTG, connective tissue graft; MTDP, modified tunnel double papilla.

### 3.2. Treatment Success Rates by Recession Category and Tooth Distribution

Treatment success was evaluated according to Miller Class (> 70% recession coverage for Miller I/II and > 50% coverage for Miller III) [8, 9] and tooth position in the arch. The results indicate successful treatment in 97.7% (*n* = 42), 74.4% (*n* = 29), and 73.9% (*n* = 17), Miller I, II/RT1, and III recessions/RT2, respectively. Medial (central) and lateral incisors and canines were defined as the anterior group of teeth and were separated from the premolar group, for convenience. The results indicate that 78.4% (*n* = 58) of teeth in the anterior group and 96.8% (*n* = 30) of teeth in the premolar group achieved successful coverage. In addition, 17 recessions (11 Miller Class I–II/RT1 and 6 Miller Class III/RT2) in 13 patients (9 and 4, respectively) underwent surgical retreatment, amounting to 17 patients in total (14 females and 3 males) who received root coverage retreatment.

### 3.3. Variables Affecting Success of Root Coverage

Among all the available data, several significant correlations were found when a logistic regression mixed model was used (Table 3). This model accounted for patients as a random effect because some patients were treated for multiple recession defects. Treatment success was negatively associated with smoking (estimate = −29.99, *p*  < 0.001) and recession type: Miller Class II/RT1 (estimate = −21.45, *p*  < 0.001) and Miller Class III/RT2 (estimate = −21.43, *p*  < 0.001). Tooth position was found to influence procedures’ outcomes. While lateral incisors were negatively associated with treatment success (−21.11, *p*  < 0.001), canines (1.71, *p*  < 0.001) and first premolars (10.31, *p*  < 0.001) were positively associated with root coverage outcomes.

**Table 3:** Variables associated with recession coverage success.

Parameter	Estimate	Std. error	BH‐corrected *p* value
Smoking	−20.996	5.64	<0.001
Miller Cl II/RT1	−21.459	0.039	<0.001
Miller Cl III/RT2	−21.439	2.821	<0.001
Lateral (vs. incisor)	−21.114	5.476	<0.001
Canine (vs. incisor)	1.711	0.035	<0.001
First premolar (vs. incisor)	10.313	0.035	<0.001

*Note:* Logistic regression mixed model. Estimate—indicates whether the parameter is negatively (−) or positively (+) associated with recession coverage success.

### 3.4. Variables Affecting Gingival Clinical Parameters

A univariate linear regression mixed model identified several associations between clinical parameters and RRD, KTG, and clinical attachment level (CAL) gain. The results indicate that:

CAL gain—RD, RW, and Miller Cl III/RT2 are positively associated with CAL gain, as evidenced by positive estimate values and *p*  < 0.05 (Table 4). Miller Class II/RT1 came close to reaching significance (*p* = 0.07). Preoperative KT width is negatively associated with CAL gain (*p* = 0.015).

**Table 4:** Variables associated with additional clinical parameters.

Variables	CAL gain			KTG			RRD		
	Estimate	CI	*p* Value	Estimate	CI	*p* Value	Estimate	CI	*p* Value
Smoking	–	–	–	−1.09	(−2.1)–(0.09)	0.039	–	–	–
Miller Cl II/RT1	0.61	(−0.05)–1.29	0.07	–	–	–	0.72	0.21–1.21	0.005
Miller Cl III/RT2	1.14	0.29–1.99	0.02	0.99	0.34–1.63	0.008	0.92	0.31–1.54	0.005
RD	0.71	0.59–0.83	<0.001	0.34	0.21–0.47	<0.001	0.28	0.16–0.4	<0.001
RW	0.65	0.29–1	<0.001	0.33	0.04–0.63	0.028	0.3	0.08–0.58	0.03
KT	−0.45	(−0.81)–(−0.09)	0.015	−0.57	(−0.84)–(0.29)	<0.001	–	–	–

*Note:* Univariate linear regression mixed model. Estimate—indicates whether the parameter is negatively (−), or positively (+) associated with surrogate clinical parameters.

Abbreviations: CAL, clinical attachment level; CI, confidence interval; KT, keratinized tissue; KTG, keratinized tissue gain; RD, recession depth; RRD, residual recession depth; RW, recession width.

KTG—RW and Miller Class III/RT2 are associated with postoperative KTG. KT and smoking, however, were negatively associated with this parameter (Table 4).

RRD—RD, RW, and both Miller Class II and III/RT1 and 2 are positively associated with RRD.

Age and sex, type of surgical technique, upper/lower jaw, and tooth position in the arch were not found to be statistically associated with CAL gain, KTG, and RRD.

## 4. Discussion

### 4.1. Initial Variables Associated With Root Coverage Success

Every treatment should be guided by a clear objective and align with the patient’s expectations. For root coverage procedures, the primary goals are functional, esthetic, and long‐term stability. Achieving CRC with seamless color blending is paramount [24]. Our findings suggest a potential negative impact of smoking on long‐term treatment success; however, this observation must be interpreted with caution due to the pronounced imbalance between smokers and nonsmokers in our sample (4 vs. 52), which limits the statistical reliability of the comparison. Nevertheless, the direction of the trend aligns with previous evidence showing that current smoking reduces the likelihood of achieving root coverage, independent of the surgical technique used [16, 25, 26]. It is also important to note that smoking intensity and duration are key factors influencing clinical outcomes [27]. For example, Andia et al. [28] reported that consuming at least 20 cigarettes per day over the preceding five years significantly impaired root coverage outcomes at 2‐year follow‐up.

Tooth position was also found to be associated with treatment outcomes. Position within the arch was found to be significant, that is, lateral incisors were negatively associated with treatment success, whereas canines and first premolars were positively associated with successful defect coverage. Position on the upper vs. lower arch, however, did not impact treatment success. This finding confirms an observation previously described in a systematic review by Zucchelli et al. [17] that canines are associated with higher rates of root coverage without any relation to maxillary or mandibular dentition. Our study confirms Zucchelli’s observation, in that maxillary or mandibular tooth position does not affect treatment success over time. The higher success rates of recession coverage on canines and premolars, in this cohort, compared with lateral incisors may be related to their thicker gingival phenotype, broader KT, more favorable root convexity, and better‐developed interdental papillae, which together enhance flap stability and vascular supply.

**Figure 1:**
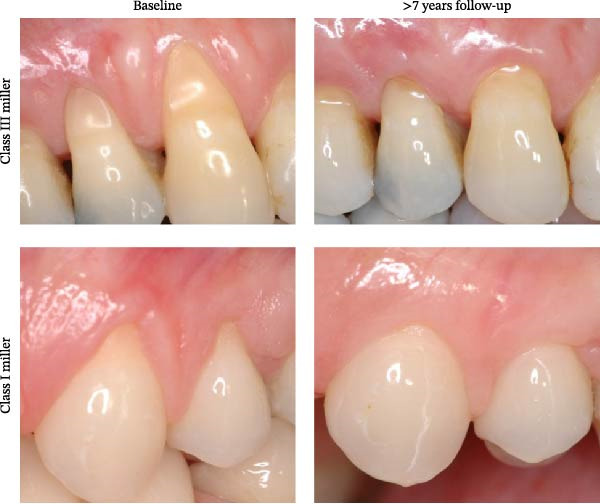
Miller recession classification and postoperative keratinized tissue gain. Representative preoperative and long‐term postoperative images of Miller Class III (top) and Class I (bottom) gingival recession defects. The Class III defect shows substantial keratinized tissue (KT) gain after 14 years, whereas the Class I defect demonstrates complete root coverage with more modest KT gain after 7 years. Although the percentage of root coverage is higher in the Class I case, the total KT gain is greater in the Class III case, consistent with the observed negative correlation between initial KT width and KT gain.

Our results also indicate that initial RD and RW values have a significant positive impact on CAL gain, KTG, and RRD. This agrees with previous findings that postsurgical RD reduction and level of root coverage are influenced by the initial status of gingival tissue, RD, and RW [29, 30]. Our results reveal that the initial width of KT is negatively correlated with KTG. This observation is largely influenced by the arithmetic effect of the calculation: defects with narrower initial KT that achieve successful root coverage exhibit greater absolute increases in KT. For example, as illustrated in Figure 1, a Class III recession defect with limited initial KT shows a more pronounced KTG over the long term than a Class I defect, despite the latter achieving complete coverage. This helps reconcile the apparent discrepancy between percentage root coverage and absolute KTG across different recession classes.

The lack of significant differences in root coverage among the surgical techniques may be explained by the fact that, when performed correctly, these approaches share similar biological principles and healing dynamics, making outcomes more dependent on patient‐ and site‐specific factors than on the choice of technique itself.

### 4.2. Methodological Variability in Assessing Recession Coverage Stability

In the mucogingival literature, there is considerable diversity in study design and methodology [31]. Regarding follow‐up periods, while some studies report only immediate postsurgical outcomes [29, 32–34], others present long‐term data, extending up to 35 years [35, 36]. In our study, the average follow‐up period was 3.5 years, with a maximum of 15 years.

Most mucogingival studies focus on a single surgical approach, such as the coronally advanced flap combined with a subepithelial connective tissue graft [37]. Only a few assess multiple techniques within the same study [31]. Our study did not restrict itself to one surgical method and found no significant superiority among the techniques used.

Additionally, the sample size in most previous studies is relatively limited, often involving up to 35 patients [29], whereas our study included 56 patients. While most mucogingival studies are conducted in academic or controlled dental school environments, only a minority report outcomes from real‐life private practice settings [29]. Our investigation was conducted in a private practice context, with rigorous statistical analyses performed in an academic institution.

Stability of the gingival margin over time is often attributed to biotype modification [38, 39]. Yet, long‐term stability is not achieved in a significant percentage of cases [9]. Similarly, Pini Prato et al. [35] observed a progressive recurrence of gingival recession in 39% of patients treated with a coronally advanced flap over a 14‐year follow‐up. In contrast, our results indicate that root coverage achieved immediately after the procedure remained stable over a 40‐month follow‐up.

### 4.3. Study Limitations and Advantages

Although the definitions of treatment success used in this study (70% and 50% root coverage for Miller Class I–II/RT1 and Miller Class III/RT2 defects, respectively) were derived from established systematic reviews and meta‐analyses [8, 10] to provide standardized reference points for analysis, they cannot fully reflect the individualized nature of clinical outcomes. In practice, the perception of success is inherently patient‐centered, with patient satisfaction representing the most meaningful determinant of treatment success. Our study includes a large range of surgical procedures, which increases heterogeneity. All the procedures employed, however, are well documented for the treatment of Miller Class II/RT1 and III/RT2 recession defects [18–23], and no meta analyses were so far able to show significant superiority of one procedure over the others that were used in this study [40–42]. The procedures were performed over a longtime span during which several surgical approaches were gradually introduced into clinical practice, and the choice of technique was therefore made by the clinician at the time of treatment based on prevailing professional standards, defect morphology, and clinical judgment. The absence of significant differences in root coverage outcomes across techniques in our dataset is consistent with the existing evidence [41, 43]. This may be explained by the fact that, when executed correctly, these procedures rely on comparable biological principles, such as tension‐free flap advancement, adequate vascular supply, and stable flap or graft positioning, which tend to standardize healing dynamics. As a result, treatment outcomes appear to depend more on patient‐ and site‐related factors than on the specific surgical approach selected. Although we found that smoking was negatively associated with treatment success, we cannot report the specific impact of smoking intensity, since these data were not collected (e.g., number of cigarettes per day). Additionally, standardized preoperative tissue thickness was not assessed, which may have influenced treatment outcomes. A further potential limitation is selection bias inherent to retrospective analyses; patients included in this study were those who maintained follow‐up care and were treated in a private practice setting, which may not fully represent the broader population. Regarding patient compliance, all patients adhered to follow‐up visits and periodontal maintenance schedules, minimizing variability related to postoperative care.

Gathering long‐term outcomes for root coverage procedures typically requires meticulous documentation and detailed statistical analysis, which are more commonly performed in academic environments. By integrating clinical records from a private practice with rigorous academic analysis, this study uniquely presents comprehensive data from real‐world clinical settings and nonacademic patients, thereby providing valuable insights into practical, everyday outcomes.

## 5. Conclusions

Root coverage success is negatively associated with smoking, Miller Class II–III/RT1−2 recessions, and a lateral tooth position. Canines and first premolars have significantly higher rates of successful treatment. CAL gain, KTG, and RRD are positively influenced by initial RD, RW, and Miller Class III/RT2. Among the surgical techniques investigated in this study, no approach demonstrated clear clinical superiority. This suggests that, when performed correctly, these procedures share similar biological principles and healing dynamics, making outcomes more dependent on patient‐ and site‐specific factors rather than on the choice of technique alone. Therefore, the selection of a surgical approach should be primarily guided by the current condition of the tissues and the clinician’s experience and proficiency with evidence‐based root coverage procedures. These findings provide practical guidance for clinicians: careful assessment of patient‐related factors, recession characteristics, and tooth position should inform treatment planning, while adherence to established surgical principles is key to achieving predictable long‐term outcomes.

## Author Contributions

Conceptualization: Michael Saminsky and Alon Sebaoun. Project administration: Alon Sebaoun and Michael Saminsky. Data curation: Liat Chaushu. Validation: Benjamin R. Coyac and Liat Chaushu. Investigation: Alon Sebaoun and Michael Saminsky. Formal analysis: Alon Sebaoun and Michael Saminsky. Analyzed the data and writing – original draft: Michael Saminsky, Alon Sebaoun, and Benjamin R. Coyac. Led the writing and writing – review and editing: all authors.

## Funding

This study did not receive any funding.

## Conflicts of Interest

The authors declare no conflicts of interest.

## Data Availability

The data that support the findings of this study are available upon request from the corresponding author. The data are not publicly available due to privacy or ethical restrictions.
